# Supplementary material for: Significance of hub genes and immune cell infiltration identified by bioinformatics analysis in pelvic organ prolapse
Source: PeerJ. 2020 Aug 18;8:e9773. doi: 10.7717/peerj.9773 (PMC7441923; doi:10.7717/peerj.9773)
Supplement: Supplemental Information 1 [file peerj-08-9773-s001.docx]

| Sample | GEM | SAT | THBS1 | TMEM70 | IFRD1 | SOD2 | CXCR4 | ZNF331 |
| --- | --- | --- | --- | --- | --- | --- | --- | --- |
| POP 1 | 12.480 | 12.816 | 9.6164 | 12.361 | 10.324 | 10.752 | 11.232 | 11.032 |
| POP 2 | 12.480 | 12.070 | 8.864 | 10.740 | 9.7602 | 11.061 | 9.862 | 9.570 |
| POP 3 | 11.660 | 12.401 | 8.3271 | 11.189 | 9.728 | 10.662 | 10.276 | 9.638 |
| POP 4 | 12.412 | 13.333 | 9.2515 | 12.036 | 10.022 | 12.464 | 10.962 | 10.869 |
| POP 5 | 11.287 | 12.502 | 9.4945 | 12.405 | 9.798 | 11.044 | 10.316 | 10.034 |
| POP 6 | 12.910 | 12.972 | 9.4288 | 11.260 | 10.465 | 11.361 | 11.034 | 9.500 |
| POP 7 | 14.020 | 13.769 | 10.051 | 12.363 | 10.908 | 12.032 | 11.475 | 11.035 |
| POP 8 | 13.171 | 13.060 | 12.428 | 12.119 | 10.119 | 12.837 | 10.341 | 12.010 |
| POP 9 | 12.435 | 12.782 | 9.011 | 11.210 | 9.8277 | 11.708 | 10.530 | 10.041 |
| POP 10 | 11.680 | 12.293 | 8.583 | 11.131 | 9.727 | 10.949 | 9.602 | 9.194 |
| POP 11 | 10.992 | 11.875 | 8.653 | 10.530 | 9.038 | 10.929 | 8.474 | 9.514 |
| POP 12 | 11.335 | 11.520 | 7.559 | 11.182 | 9.252 | 10.660 | 7.637 | 8.938 |
| non-POP 1 | 11.886 | 12.553 | 8.678 | 11.165 | 9.535 | 10.337 | 10.473 | 9.356 |
| non-POP 2 | 11.930 | 12.426 | 8.431 | 11.016 | 9.667 | 10.427 | 10.063 | 9.508 |
| non-POP 3 | 10.732 | 11.769 | 8.599 | 11.101 | 9.014 | 10.284 | 9.2559 | 9.172 |
| non-POP 4 | 11.749 | 12.324 | 8.229 | 11.038 | 9.417 | 10.611 | 10.107 | 9.491 |
| non-POP 5 | 10.751 | 12.333 | 8.331 | 11.439 | 9.281 | 10.519 | 10.479 | 9.428 |
| non-POP 6 | 12.222 | 12.456 | 9.053 | 10.969 | 9.566 | 10.874 | 9.2017 | 9.037 |
| non-POP 7 | 12.450 | 12.595 | 8.313 | 11.470 | 9.423 | 10.586 | 9.2462 | 9.431 |
| non-POP 8 | 11.699 | 12.191 | 9.537 | 10.559 | 9.312 | 11.270 | 9.491 | 9.394 |
| non-POP 9 | 12.203 | 12.523 | 8.677 | 11.043 | 9.492 | 10.885 | 10.637 | 9.321 |
| non-POP 10 | 11.52173 | 12.03621 | 8.738375 | 11.35099 | 9.043839 | 10.98532 | 10.92946 | 8.913186 |
| non-POP 11 | 11.08233 | 11.87018 | 8.846435 | 10.52347 | 9.312258 | 10.74899 | 9.206005 | 9.625056 |
| non-POP 12 | 11.69084 | 11.4911 | 7.545446 | 10.94393 | 9.314631 | 10.74466 | 7.632972 | 8.820101 |
