# Supplementary material for: Significance of hub genes and immune cell infiltration identified by bioinformatics analysis in pelvic organ prolapse
Source: PeerJ. 2020 Aug 18;8:e9773. doi: 10.7717/peerj.9773 (PMC7441923; doi:10.7717/peerj.9773)
Supplement: Supplemental Information 2 [file peerj-08-9773-s002.docx]

| Group | age（year） | BMI | Number of deliveries |
| --- | --- | --- | --- |
|  |  |  |  |
| The number of patients  （*n*=12） | 57.21±12.11 | 23.97±3.03 | 1.27±0.89 |
