# Supplementary material for: Significance of hub genes and immune cell infiltration identified by bioinformatics analysis in pelvic organ prolapse
Source: PeerJ. 2020 Aug 18;8:e9773. doi: 10.7717/peerj.9773 (PMC7441923; doi:10.7717/peerj.9773)
Supplement: Supplemental Information 3 [file peerj-08-9773-s003.docx]

| Immune cells |
| --- |
| B.cells.naive |
| B.cells.memory |
| Plasma.cells |
| T.cells.CD8 |
| T.cells.CD4.memory.resting |
| T.cells.CD4.memory.activated |
| T.cells.follicular.helper |
| T.cells.regulatory..Tregs. |
| T.cells.gamma.delta |
| NK.cells.resting |
| NK.cells.activated |
| Monocytes |
| Macrophages.M0 |
| Macrophages.M1 |
| Macrophages.M2 |
| Dendritic.cells.resting |
| Dendritic.cells.activated |
| Mast.cells.resting |
| Mast.cells.activated |
| Eosinophils |
| Neutrophils |
