# Supplementary material for: Significance of hub genes and immune cell infiltration identified by bioinformatics analysis in pelvic organ prolapse
Source: PeerJ. 2020 Aug 18;8:e9773. doi: 10.7717/peerj.9773 (PMC7441923; doi:10.7717/peerj.9773)
Supplement: Supplemental Information 4 [file peerj-08-9773-s004.docx]

| Gene | forward primer | reverse primer |
| --- | --- | --- |
| IFRD1 | 5'‐TGCAGTGGTTATAGCGATCCT‐3' | 5'‐CCTTGTCTTCGCACTCTTATCC‐3' |
| ZNF331 | 5'‐TTCGCCGACGTAGCCATAGA‐3' | 5'‐CGTCCCAGTACAGGTCCCT‐3' |
| THBS1 | 5'‐AGACTCCGCATCGCAAAGG‐3' | 5'‐TCACCACGTTGTTGTCAAGGG‐3' |
| FLJ20533/TMEM70 | 5'‐CGAGCGCAGATCCCTGTTTAT‐3' | 5'‐CTCGGGCCATATTGCCAGTAT‐3' |
| CXCR4 | 5'‐ACTACACCGAGGAAATGGGCT‐3' | 5'‐CCCACAATGCCAGTTAAGAAGA‐3' |
| GEM | 5'‐GCAACCGCCATTCTGCTAC‐3' | 5'‐CTCCCCTATGAGCACCACTC‐3' |
| SOD2 | 5'‐GCTCCGGTTTTGGGGTATCTG‐3' | 5'‐GCGTTGATGTGAGGTTCCAG‐3' |
| SAT | 5'‐TCTGGCATTGAGTCTCTGCG‐3' | 5'‐AGGAGCCATAGGTGGAATCAG‐3' |
| GAPDH | 5'‐ACAACTTTGGTATCGTGGAAGG‐3' | 5'‐GCCATCACGCCACAGTTTC‐3' |
