# Supplementary material for: Significance of hub genes and immune cell infiltration identified by bioinformatics analysis in pelvic organ prolapse
Source: PeerJ. 2020 Aug 18;8:e9773. doi: 10.7717/peerj.9773 (PMC7441923; doi:10.7717/peerj.9773)
Supplement: Supplemental Information 5 [file peerj-08-9773-s005.docx]

| ID | Description | pvalue | p.adjust | qvalue | geneID Count |
| --- | --- | --- | --- | --- | --- |
| GO:0062023 | collagen-containing extracellular matrix | 2.62E-07 | 4.12E-05 | 3.01E-05 | 10 |
| GO:0050727 | regulation of inflammatory response | 3.26E-07 | 0.000342 | 0.000241 | 11 |
| GO:0002446 | neutrophil mediated immunity | 4.90E-07 | 0.000342 | 0.000241 | 11 |
| GO:0002237 | response to molecule of bacterial origin | 6.11E-07 | 0.000342 | 0.000241 | 9 |
| GO:0032103 | positive regulation of response to external stimulus | 7.17E-07 | 0.000342 | 0.000241 | 9 |
| GO:0031012 | extracellular matrix | 1.12E-06 | 8.82E-05 | 6.45E-05 | 10 |
| GO:0060326 | cell chemotaxis | 1.48E-06 | 0.000522 | 0.000367 | 8 |
| GO:0002683 | negative regulation of immune system process | 1.64E-06 | 0.000522 | 0.000367 | 10 |
| GO:0050900 | leukocyte migration | 2.24E-06 | 0.000573 | 0.000404 | 10 |
| GO:0030595 | leukocyte chemotaxis | 2.40E-06 | 0.000573 | 0.000404 | 7 |
| GO:0043312 | neutrophil degranulation | 3.14E-06 | 0.000619 | 0.000436 | 10 |
| GO:0002283 | neutrophil activation involved in immune response | 3.32E-06 | 0.000619 | 0.000436 | 10 |
| GO:0002694 | regulation of leukocyte activation | 3.57E-06 | 0.000619 | 0.000436 | 10 |
| GO:0042119 | neutrophil activation | 4.04E-06 | 0.000643 | 0.000453 | 10 |
| GO:0032496 | response to lipopolysaccharide | 4.62E-06 | 0.000678 | 0.000478 | 8 |
| GO:0050786 | RAGE receptor binding | 5.82E-06 | 0.001245 | 0.000906 | 3 |
| GO:0061041 | regulation of wound healing | 6.70E-06 | 0.000914 | 0.000644 | 6 |
| GO:0050729 | positive regulation of inflammatory response | 9.88E-06 | 0.001257 | 0.000886 | 6 |
| GO:0002523 | leukocyte migration involved in inflammatory response | 1.14E-05 | 0.001365 | 0.000961 | 3 |
| GO:0032652 | regulation of interleukin-1 production | 1.46E-05 | 0.001637 | 0.001153 | 5 |
| GO:0097529 | myeloid leukocyte migration | 1.80E-05 | 0.001841 | 0.001297 | 6 |
| GO:0048660 | regulation of smooth muscle cell proliferation | 1.92E-05 | 0.001841 | 0.001297 | 6 |
| GO:1903034 | regulation of response to wounding | 1.99E-05 | 0.001841 | 0.001297 | 6 |
| GO:0048659 | smooth muscle cell proliferation | 2.05E-05 | 0.001841 | 0.001297 | 6 |
| GO:0050711 | negative regulation of interleukin-1 secretion | 2.12E-05 | 0.001841 | 0.001297 | 3 |
| GO:0002791 | regulation of peptide secretion | 2.30E-05 | 0.001912 | 0.001346 | 9 |
| GO:0032612 | interleukin-1 production | 2.58E-05 | 0.002053 | 0.001446 | 5 |
| GO:0097193 | intrinsic apoptotic signaling pathway | 3.00E-05 | 0.00221 | 0.001557 | 7 |
| GO:0002544 | chronic inflammatory response | 3.01E-05 | 0.00221 | 0.001557 | 3 |
| GO:1904950 | negative regulation of establishment of protein localization | 3.68E-05 | 0.002601 | 0.001832 | 6 |
| GO:0090303 | positive regulation of wound healing | 3.94E-05 | 0.00269 | 0.001895 | 4 |
| GO:0034774 | secretory granule lumen | 4.67E-05 | 0.001966 | 0.001437 | 7 |
| GO:1903531 | negative regulation of secretion by cell | 4.74E-05 | 0.003124 | 0.0022 | 6 |
| GO:0002696 | positive regulation of leukocyte activation | 5.53E-05 | 0.003524 | 0.002482 | 7 |
| GO:0060205 | cytoplasmic vesicle lumen | 6.47E-05 | 0.001966 | 0.001437 | 7 |
| GO:0031983 | vesicle lumen | 6.59E-05 | 0.001966 | 0.001437 | 7 |
| GO:0071674 | mononuclear cell migration | 6.90E-05 | 0.004248 | 0.002992 | 4 |
| GO:0002218 | activation of innate immune response | 7.34E-05 | 0.004248 | 0.002992 | 7 |
| GO:0050867 | positive regulation of cell activation | 7.34E-05 | 0.004248 | 0.002992 | 7 |
| GO:0050709 | negative regulation of protein secretion | 8.05E-05 | 0.00444 | 0.003127 | 5 |
| GO:1903036 | positive regulation of response to wounding | 8.18E-05 | 0.00444 | 0.003127 | 4 |
| GO:0050710 | negative regulation of cytokine secretion | 8.64E-05 | 0.00444 | 0.003127 | 4 |
| GO:0007159 | leukocyte cell-cell adhesion | 8.74E-05 | 0.00444 | 0.003127 | 7 |
| GO:0051048 | negative regulation of secretion | 8.83E-05 | 0.00444 | 0.003127 | 6 |
| GO:0098552 | side of membrane | 9.43E-05 | 0.001966 | 0.001437 | 7 |
| GO:0002792 | negative regulation of peptide secretion | 9.54E-05 | 0.00467 | 0.003289 | 5 |
| GO:0005766 | primary lysosome | 0.0001 | 0.001966 | 0.001437 | 5 |
| GO:0042582 | azurophil granule | 0.0001 | 0.001966 | 0.001437 | 5 |
| GO:0050708 | regulation of protein secretion | 0.000104 | 0.004971 | 0.003501 | 8 |
| GO:0002224 | toll-like receptor signaling pathway | 0.000112 | 0.005229 | 0.003683 | 5 |
| GO:0032102 | negative regulation of response to external stimulus | 0.000115 | 0.005247 | 0.003696 | 7 |
| GO:0033002 | muscle cell proliferation | 0.000131 | 0.00584 | 0.004113 | 6 |
| GO:0071621 | granulocyte chemotaxis | 0.000157 | 0.006833 | 0.004813 | 4 |
| GO:2001242 | regulation of intrinsic apoptotic signaling pathway | 0.000182 | 0.007731 | 0.005445 | 5 |
| GO:1904035 | regulation of epithelial cell apoptotic process | 0.000188 | 0.007824 | 0.00551 | 4 |
| GO:0045089 | positive regulation of innate immune response | 0.000193 | 0.007863 | 0.005538 | 7 |
| GO:0043394 | proteoglycan binding | 0.000237 | 0.025376 | 0.018474 | 3 |
| GO:1904037 | positive regulation of epithelial cell apoptotic process | 0.000251 | 0.009977 | 0.007027 | 3 |
| GO:0071222 | cellular response to lipopolysaccharide | 0.00026 | 0.010139 | 0.007141 | 5 |
| GO:0001558 | regulation of cell growth | 0.000279 | 0.010544 | 0.007426 | 7 |
| GO:2001233 | regulation of apoptotic signaling pathway | 0.000283 | 0.010544 | 0.007426 | 7 |
| GO:0032692 | negative regulation of interleukin-1 production | 0.000292 | 0.010544 | 0.007426 | 3 |
| GO:0051249 | regulation of lymphocyte activation | 0.000301 | 0.010544 | 0.007426 | 7 |
| GO:0097530 | granulocyte migration | 0.000308 | 0.010544 | 0.007426 | 4 |
| GO:0070555 | response to interleukin-1 | 0.000311 | 0.010544 | 0.007426 | 5 |
| GO:0071219 | cellular response to molecule of bacterial origin | 0.000311 | 0.010544 | 0.007426 | 5 |
| GO:0014002 | astrocyte development | 0.000315 | 0.010544 | 0.007426 | 3 |
| GO:0001818 | negative regulation of cytokine production | 0.000341 | 0.011136 | 0.007843 | 6 |
| GO:0050866 | negative regulation of cell activation | 0.000344 | 0.011136 | 0.007843 | 5 |
| GO:0051224 | negative regulation of protein transport | 0.000361 | 0.011498 | 0.008099 | 5 |
| GO:0002548 | monocyte chemotaxis | 0.000388 | 0.012157 | 0.008562 | 3 |
| GO:0046879 | hormone secretion | 0.000416 | 0.012818 | 0.009028 | 6 |
| GO:1901342 | regulation of vasculature development | 0.000427 | 0.012932 | 0.009108 | 7 |
| GO:0002221 | pattern recognition receptor signaling pathway | 0.000437 | 0.013002 | 0.009158 | 5 |
| GO:0050864 | regulation of B cell activation | 0.000442 | 0.013002 | 0.009158 | 4 |
| GO:1904019 | epithelial cell apoptotic process | 0.000458 | 0.013252 | 0.009334 | 4 |
| GO:0035325 | Toll-like receptor binding | 0.00049 | 0.034976 | 0.025462 | 2 |
| GO:0009914 | hormone transport | 0.000495 | 0.0141 | 0.009931 | 6 |
| GO:0045088 | regulation of innate immune response | 0.000525 | 0.014737 | 0.010379 | 7 |
| GO:0071216 | cellular response to biotic stimulus | 0.000535 | 0.014817 | 0.010436 | 5 |
| GO:0032873 | negative regulation of stress-activated MAPK cascade | 0.000567 | 0.015246 | 0.010738 | 3 |
| GO:0070303 | negative regulation of stress-activated protein kinase signaling cascade | 0.000567 | 0.015246 | 0.010738 | 3 |
| GO:0072594 | establishment of protein localization to organelle | 0.000615 | 0.016102 | 0.011341 | 7 |
| GO:0050704 | regulation of interleukin-1 secretion | 0.000636 | 0.016102 | 0.011341 | 3 |
| GO:0070663 | regulation of leukocyte proliferation | 0.000636 | 0.016102 | 0.011341 | 5 |
| GO:0042326 | negative regulation of phosphorylation | 0.00064 | 0.016102 | 0.011341 | 7 |
| GO:0015867 | ATP transport | 0.000666 | 0.016102 | 0.011341 | 2 |
| GO:0045779 | negative regulation of bone resorption | 0.000666 | 0.016102 | 0.011341 | 2 |
| GO:0051238 | sequestering of metal ion | 0.000666 | 0.016102 | 0.011341 | 2 |
| GO:0070486 | leukocyte aggregation | 0.000666 | 0.016102 | 0.011341 | 2 |
| GO:0009266 | response to temperature stimulus | 0.000706 | 0.016858 | 0.011874 | 5 |
| GO:0016049 | cell growth | 0.000737 | 0.017048 | 0.012008 | 7 |
| GO:0031663 | lipopolysaccharide-mediated signaling pathway | 0.00075 | 0.017048 | 0.012008 | 3 |
| GO:2000116 | regulation of cysteine-type endopeptidase activity | 0.000751 | 0.017048 | 0.012008 | 5 |
| GO:0005770 | late endosome | 0.000753 | 0.013128 | 0.009594 | 5 |
| GO:1903706 | regulation of hemopoiesis | 0.000756 | 0.017048 | 0.012008 | 7 |
| GO:2001234 | negative regulation of apoptotic signaling pathway | 0.000766 | 0.017048 | 0.012008 | 5 |
| GO:0051917 | regulation of fibrinolysis | 0.000785 | 0.017048 | 0.012008 | 2 |
| GO:0061888 | regulation of astrocyte activation | 0.000785 | 0.017048 | 0.012008 | 2 |
| GO:0070431 | nucleotide-binding oligomerization domain containing 2 signaling pathway | 0.000785 | 0.017048 | 0.012008 | 2 |
| GO:0071900 | regulation of protein serine/threonine kinase activity | 0.000866 | 0.018581 | 0.013087 | 7 |
| GO:0046851 | negative regulation of bone remodeling | 0.000914 | 0.018911 | 0.013319 | 2 |
| GO:0050713 | negative regulation of interleukin-1 beta secretion | 0.000914 | 0.018911 | 0.013319 | 2 |
| GO:0061042 | vascular wound healing | 0.000914 | 0.018911 | 0.013319 | 2 |
| GO:2000351 | regulation of endothelial cell apoptotic process | 0.000921 | 0.018911 | 0.013319 | 3 |
| GO:0002521 | leukocyte differentiation | 0.000931 | 0.018918 | 0.013325 | 7 |
| GO:2001056 | positive regulation of cysteine-type endopeptidase activity | 0.000955 | 0.019041 | 0.013411 | 4 |
| GO:0050701 | interleukin-1 secretion | 0.000967 | 0.019041 | 0.013411 | 3 |
| GO:1905953 | negative regulation of lipid localization | 0.000967 | 0.019041 | 0.013411 | 3 |
| GO:0036041 | long-chain fatty acid binding | 0.000983 | 0.048441 | 0.035264 | 2 |
| GO:0017014 | protein nitrosylation | 0.001053 | 0.020315 | 0.014308 | 2 |
| GO:0018119 | peptidyl-cysteine S-nitrosylation | 0.001053 | 0.020315 | 0.014308 | 2 |
| GO:2001244 | positive regulation of intrinsic apoptotic signaling pathway | 0.001064 | 0.02032 | 0.014312 | 3 |
| GO:0097237 | cellular response to toxic substance | 0.001084 | 0.020504 | 0.014441 | 5 |
| GO:0009636 | response to toxic substance | 0.001099 | 0.020571 | 0.014489 | 7 |
| GO:0030169 | low-density lipoprotein particle binding | 0.001132 | 0.048441 | 0.035264 | 2 |
| GO:0001780 | neutrophil homeostasis | 0.001201 | 0.021791 | 0.015348 | 2 |
| GO:0051503 | adenine nucleotide transport | 0.001201 | 0.021791 | 0.015348 | 2 |
| GO:0010212 | response to ionizing radiation | 0.001214 | 0.021791 | 0.015348 | 4 |
| GO:0072577 | endothelial cell apoptotic process | 0.00122 | 0.021791 | 0.015348 | 3 |
| GO:0052548 | regulation of endopeptidase activity | 0.001221 | 0.021791 | 0.015348 | 6 |
| GO:0043030 | regulation of macrophage activation | 0.001275 | 0.022357 | 0.015747 | 3 |
| GO:0045637 | regulation of myeloid cell differentiation | 0.001276 | 0.022357 | 0.015747 | 5 |
| GO:0036019 | endolysosome | 0.00131 | 0.020564 | 0.015029 | 2 |
| GO:0007249 | I-kappaB kinase/NF-kappaB signaling | 0.001345 | 0.02334 | 0.016439 | 5 |
| GO:0015868 | purine ribonucleotide transport | 0.001358 | 0.02334 | 0.016439 | 2 |
| GO:0046883 | regulation of hormone secretion | 0.001369 | 0.02334 | 0.016439 | 5 |
| GO:0048662 | negative regulation of smooth muscle cell proliferation | 0.00145 | 0.024144 | 0.017005 | 3 |
| GO:1901264 | carbohydrate derivative transport | 0.00145 | 0.024144 | 0.017005 | 3 |
| GO:0045765 | regulation of angiogenesis | 0.001456 | 0.024144 | 0.017005 | 6 |
| GO:0051251 | positive regulation of lymphocyte activation | 0.001466 | 0.024144 | 0.017005 | 5 |
| GO:1903707 | negative regulation of hemopoiesis | 0.001481 | 0.024182 | 0.017032 | 4 |
| GO:0006882 | cellular zinc ion homeostasis | 0.001525 | 0.024231 | 0.017067 | 2 |
| GO:0010888 | negative regulation of lipid storage | 0.001525 | 0.024231 | 0.017067 | 2 |
| GO:0015865 | purine nucleotide transport | 0.001525 | 0.024231 | 0.017067 | 2 |
| GO:0043062 | extracellular structure organization | 0.001535 | 0.024231 | 0.017067 | 6 |
| GO:0071347 | cellular response to interleukin-1 | 0.001629 | 0.025458 | 0.01793 | 4 |
| GO:0002532 | production of molecular mediator involved in inflammatory response | 0.00164 | 0.025458 | 0.01793 | 3 |
| GO:0032495 | response to muramyl dipeptide | 0.001701 | 0.025458 | 0.01793 | 2 |
| GO:0034104 | negative regulation of tissue remodeling | 0.001701 | 0.025458 | 0.01793 | 2 |
| GO:0035458 | cellular response to interferon-beta | 0.001701 | 0.025458 | 0.01793 | 2 |
| GO:1902105 | regulation of leukocyte differentiation | 0.001705 | 0.025458 | 0.01793 | 5 |
| GO:0061045 | negative regulation of wound healing | 0.001706 | 0.025458 | 0.01793 | 3 |
| GO:0005775 | vacuolar lumen | 0.00172 | 0.024543 | 0.017936 | 4 |
| GO:0052547 | regulation of peptidase activity | 0.001769 | 0.026195 | 0.01845 | 6 |
| GO:0070661 | leukocyte proliferation | 0.00179 | 0.026304 | 0.018527 | 5 |
| GO:0010950 | positive regulation of endopeptidase activity | 0.001829 | 0.026666 | 0.018781 | 4 |
| GO:0055069 | zinc ion homeostasis | 0.001886 | 0.027285 | 0.019217 | 2 |
| GO:0002695 | negative regulation of leukocyte activation | 0.001913 | 0.027475 | 0.019352 | 4 |
| GO:0045685 | regulation of glial cell differentiation | 0.001988 | 0.02809 | 0.019785 | 3 |
| GO:0048708 | astrocyte differentiation | 0.001988 | 0.02809 | 0.019785 | 3 |
| GO:0034614 | cellular response to reactive oxygen species | 0.002 | 0.02809 | 0.019785 | 4 |
| GO:0030307 | positive regulation of cell growth | 0.002045 | 0.028506 | 0.020077 | 4 |
| GO:0022617 | extracellular matrix disassembly | 0.002063 | 0.028554 | 0.020111 | 3 |
| GO:0030099 | myeloid cell differentiation | 0.00208 | 0.028579 | 0.020129 | 6 |
| GO:0001933 | negative regulation of protein phosphorylation | 0.00221 | 0.030147 | 0.021234 | 6 |
| GO:0034612 | response to tumor necrosis factor | 0.002232 | 0.03023 | 0.021292 | 5 |
| GO:0042063 | gliogenesis | 0.002266 | 0.030478 | 0.021467 | 5 |
| GO:0019430 | removal of superoxide radicals | 0.002283 | 0.030494 | 0.021478 | 2 |
| GO:0048511 | rhythmic process | 0.002336 | 0.03098 | 0.02182 | 5 |
| GO:0032651 | regulation of interleukin-1 beta production | 0.00238 | 0.031347 | 0.022079 | 3 |
| GO:0050673 | epithelial cell proliferation | 0.002459 | 0.031791 | 0.022391 | 6 |
| GO:0000079 | regulation of cyclin-dependent protein serine/threonine kinase activity | 0.002463 | 0.031791 | 0.022391 | 3 |
| GO:0046916 | cellular transition metal ion homeostasis | 0.002463 | 0.031791 | 0.022391 | 3 |
| GO:0060055 | angiogenesis involved in wound healing | 0.002495 | 0.031987 | 0.022529 | 2 |
| GO:0001776 | leukocyte homeostasis | 0.002549 | 0.032454 | 0.022858 | 3 |
| GO:0035578 | azurophil granule lumen | 0.002573 | 0.031212 | 0.02281 | 3 |
| GO:0010008 | endosome membrane | 0.002584 | 0.031212 | 0.02281 | 6 |
| GO:0016485 | protein processing | 0.00263 | 0.032596 | 0.022958 | 5 |
| GO:2001235 | positive regulation of apoptotic signaling pathway | 0.002633 | 0.032596 | 0.022958 | 4 |
| GO:0034599 | cellular response to oxidative stress | 0.002669 | 0.032596 | 0.022958 | 5 |
| GO:0023061 | signal release | 0.002698 | 0.032596 | 0.022958 | 6 |
| GO:0006862 | nucleotide transport | 0.002717 | 0.032596 | 0.022958 | 2 |
| GO:0048143 | astrocyte activation | 0.002717 | 0.032596 | 0.022958 | 2 |
| GO:0071450 | cellular response to oxygen radical | 0.002717 | 0.032596 | 0.022958 | 2 |
| GO:0071451 | cellular response to superoxide | 0.002717 | 0.032596 | 0.022958 | 2 |
| GO:0032602 | chemokine production | 0.002725 | 0.032596 | 0.022958 | 3 |
| GO:0010952 | positive regulation of peptidase activity | 0.002741 | 0.032596 | 0.022958 | 4 |
| GO:0002758 | innate immune response-activating signal transduction | 0.002748 | 0.032596 | 0.022958 | 5 |
| GO:1903035 | negative regulation of response to wounding | 0.002816 | 0.032996 | 0.02324 | 3 |
| GO:1904029 | regulation of cyclin-dependent protein kinase activity | 0.002816 | 0.032996 | 0.02324 | 3 |
| GO:1900407 | regulation of cellular response to oxidative stress | 0.002909 | 0.033106 | 0.023317 | 3 |
| GO:1904705 | regulation of vascular smooth muscle cell proliferation | 0.002909 | 0.033106 | 0.023317 | 3 |
| GO:1990874 | vascular smooth muscle cell proliferation | 0.002909 | 0.033106 | 0.023317 | 3 |
| GO:0030194 | positive regulation of blood coagulation | 0.002947 | 0.033106 | 0.023317 | 2 |
| GO:0042730 | fibrinolysis | 0.002947 | 0.033106 | 0.023317 | 2 |
| GO:1900048 | positive regulation of hemostasis | 0.002947 | 0.033106 | 0.023317 | 2 |
| GO:2000353 | positive regulation of endothelial cell apoptotic process | 0.002947 | 0.033106 | 0.023317 | 2 |
| GO:0009897 | external side of plasma membrane | 0.00298 | 0.033414 | 0.02442 | 4 |
| GO:0002685 | regulation of leukocyte migration | 0.003085 | 0.034253 | 0.024125 | 4 |
| GO:0050920 | regulation of chemotaxis | 0.003085 | 0.034253 | 0.024125 | 4 |
| GO:0007263 | nitric oxide mediated signal transduction | 0.003186 | 0.034768 | 0.024488 | 2 |
| GO:0050820 | positive regulation of coagulation | 0.003186 | 0.034768 | 0.024488 | 2 |
| GO:1902175 | regulation of oxidative stress-induced intrinsic apoptotic signaling pathway | 0.003186 | 0.034768 | 0.024488 | 2 |
| GO:0045638 | negative regulation of myeloid cell differentiation | 0.003298 | 0.035794 | 0.025211 | 3 |
| GO:0032611 | interleukin-1 beta production | 0.003401 | 0.036634 | 0.025802 | 3 |
| GO:0000303 | response to superoxide | 0.003433 | 0.036634 | 0.025802 | 2 |
| GO:0035456 | response to interferon-beta | 0.003433 | 0.036634 | 0.025802 | 2 |
| GO:0005769 | early endosome | 0.003467 | 0.036293 | 0.026523 | 5 |
| GO:0015748 | organophosphate ester transport | 0.003505 | 0.037188 | 0.026192 | 3 |
| GO:0000305 | response to oxygen radical | 0.00369 | 0.037993 | 0.02676 | 2 |
| GO:0010165 | response to X-ray | 0.00369 | 0.037993 | 0.02676 | 2 |
| GO:0030511 | positive regulation of transforming growth factor beta receptor signaling pathway | 0.00369 | 0.037993 | 0.02676 | 2 |
| GO:0050869 | negative regulation of B cell activation | 0.00369 | 0.037993 | 0.02676 | 2 |
| GO:1903846 | positive regulation of cellular response to transforming growth factor beta stimulus | 0.00369 | 0.037993 | 0.02676 | 2 |
| GO:0042116 | macrophage activation | 0.003719 | 0.037993 | 0.02676 | 3 |
| GO:0043281 | regulation of cysteine-type endopeptidase activity involved in apoptotic process | 0.00372 | 0.037993 | 0.02676 | 4 |
| GO:1902882 | regulation of response to oxidative stress | 0.003829 | 0.038896 | 0.027396 | 3 |
| GO:0050870 | positive regulation of T cell activation | 0.003857 | 0.038975 | 0.027451 | 4 |
| GO:0050670 | regulation of lymphocyte proliferation | 0.003926 | 0.039074 | 0.027521 | 4 |
| GO:0019058 | viral life cycle | 0.003928 | 0.039074 | 0.027521 | 5 |
| GO:0045861 | negative regulation of proteolysis | 0.003928 | 0.039074 | 0.027521 | 5 |
| GO:0032944 | regulation of mononuclear cell proliferation | 0.003997 | 0.039246 | 0.027642 | 4 |
| GO:0098869 | cellular oxidant detoxification | 0.004054 | 0.039246 | 0.027642 | 3 |
| GO:1902106 | negative regulation of leukocyte differentiation | 0.004054 | 0.039246 | 0.027642 | 3 |
| GO:0002573 | myeloid leukocyte differentiation | 0.004068 | 0.039246 | 0.027642 | 4 |
| GO:0007623 | circadian rhythm | 0.004068 | 0.039246 | 0.027642 | 4 |
| GO:0050707 | regulation of cytokine secretion | 0.004068 | 0.039246 | 0.027642 | 4 |
| GO:0032414 | positive regulation of ion transmembrane transporter activity | 0.00417 | 0.039826 | 0.02805 | 3 |
| GO:2001237 | negative regulation of extrinsic apoptotic signaling pathway | 0.00417 | 0.039826 | 0.02805 | 3 |
| GO:0002828 | regulation of type 2 immune response | 0.004228 | 0.040148 | 0.028277 | 2 |
| GO:0002042 | cell migration involved in sprouting angiogenesis | 0.004288 | 0.040148 | 0.028277 | 3 |
| GO:0048661 | positive regulation of smooth muscle cell proliferation | 0.004288 | 0.040148 | 0.028277 | 3 |
| GO:0070498 | interleukin-1-mediated signaling pathway | 0.004288 | 0.040148 | 0.028277 | 3 |
| GO:0030198 | extracellular matrix organization | 0.004466 | 0.041397 | 0.029157 | 5 |
| GO:0018198 | peptidyl-cysteine modification | 0.004511 | 0.041397 | 0.029157 | 2 |
| GO:0035767 | endothelial cell chemotaxis | 0.004511 | 0.041397 | 0.029157 | 2 |
| GO:1903978 | regulation of microglial cell activation | 0.004511 | 0.041397 | 0.029157 | 2 |
| GO:0006606 | protein import into nucleus | 0.00453 | 0.041397 | 0.029157 | 3 |
| GO:0002688 | regulation of leukocyte chemotaxis | 0.00478 | 0.043267 | 0.030474 | 3 |
| GO:0055076 | transition metal ion homeostasis | 0.00478 | 0.043267 | 0.030474 | 3 |
| GO:0051701 | interaction with host | 0.004831 | 0.043525 | 0.030656 | 4 |
| GO:0010001 | glial cell differentiation | 0.004912 | 0.044048 | 0.031024 | 4 |
| GO:1990748 | cellular detoxification | 0.005038 | 0.044964 | 0.031669 | 3 |
| GO:1903039 | positive regulation of leukocyte cell-cell adhesion | 0.005077 | 0.045103 | 0.031767 | 4 |
| GO:0045736 | negative regulation of cyclin-dependent protein serine/threonine kinase activity | 0.005101 | 0.045103 | 0.031767 | 2 |
| GO:0042113 | B cell activation | 0.005161 | 0.045297 | 0.031904 | 4 |
| GO:0032411 | positive regulation of transporter activity | 0.00517 | 0.045297 | 0.031904 | 3 |
| GO:0018149 | peptide cross-linking | 0.005408 | 0.04653 | 0.032772 | 2 |
| GO:0032691 | negative regulation of interleukin-1 beta production | 0.005408 | 0.04653 | 0.032772 | 2 |
| GO:0034142 | toll-like receptor 4 signaling pathway | 0.005408 | 0.04653 | 0.032772 | 2 |
| GO:1904030 | negative regulation of cyclin-dependent protein kinase activity | 0.005408 | 0.04653 | 0.032772 | 2 |
| GO:0021782 | glial cell development | 0.005579 | 0.047125 | 0.033192 | 3 |
| GO:0019882 | antigen processing and presentation | 0.005595 | 0.047125 | 0.033192 | 4 |
| GO:0032872 | regulation of stress-activated MAPK cascade | 0.005595 | 0.047125 | 0.033192 | 4 |
| GO:0051604 | protein maturation | 0.005632 | 0.047125 | 0.033192 | 5 |
| GO:0030224 | monocyte differentiation | 0.005724 | 0.047125 | 0.033192 | 2 |
| GO:0042092 | type 2 immune response | 0.005724 | 0.047125 | 0.033192 | 2 |
| GO:0048246 | macrophage chemotaxis | 0.005724 | 0.047125 | 0.033192 | 2 |
| GO:0048710 | regulation of astrocyte differentiation | 0.005724 | 0.047125 | 0.033192 | 2 |
| GO:0070423 | nucleotide-binding oligomerization domain containing signaling pathway | 0.005724 | 0.047125 | 0.033192 | 2 |
| GO:1903131 | mononuclear cell differentiation | 0.005724 | 0.047125 | 0.033192 | 2 |
| GO:0070302 | regulation of stress-activated protein kinase signaling cascade | 0.005775 | 0.04734 | 0.033343 | 4 |
| GO:0098754 | detoxification | 0.005862 | 0.047849 | 0.033701 | 3 |
| GO:0043122 | regulation of I-kappaB kinase/NF-kappaB signaling | 0.005959 | 0.048432 | 0.034112 | 4 |
| GO:0042752 | regulation of circadian rhythm | 0.006007 | 0.048615 | 0.034241 | 3 |
| GO:0035872 | nucleotide-binding domain, leucine rich repeat containing receptor signaling pathway | 0.006048 | 0.048744 | 0.034331 | 2 |
