# Supplementary material for: Significance of hub genes and immune cell infiltration identified by bioinformatics analysis in pelvic organ prolapse
Source: PeerJ. 2020 Aug 18;8:e9773. doi: 10.7717/peerj.9773 (PMC7441923; doi:10.7717/peerj.9773)
Supplement: Supplemental Information 6 [file peerj-08-9773-s006.docx]

| ID | Description | pvalue | p.adjust | qvalue | Count |
| --- | --- | --- | --- | --- | --- |
| hsa04657 | IL-17 signaling pathway | 3.15E-07 | 3.25E-05 | 2.69E-05 | 7 |
| hsa04668 | TNF signaling pathway | 1.64E-05 | 0.000847 | 0.000701 | 6 |
| hsa04621 | NOD-like receptor signaling pathway | 0.000241 | 0.008268 | 0.006845 | 6 |
| hsa04061 | Viral protein interaction with cytokine and cytokine receptor | 0.001464 | 0.030164 | 0.02497 | 4 |
| hsa04933 | AGE-RAGE signaling pathway in diabetic complications | 0.001464 | 0.030164 | 0.02497 | 4 |
